# Supplementary material for: Effect of a Fiber D-Limonene-Enriched Food Supplement on Intestinal Microbiota and Metabolic Parameters of Mice on a High-Fat Diet
Source: Pharmaceutics. 2021 Oct 21;13(11):1753. doi: 10.3390/pharmaceutics13111753 (PMC8620497; doi:10.3390/pharmaceutics13111753)
Supplement: Supplementary file 1 [file pharmaceutics-13-01753-s001.zip › pharmaceutics-1396599-supplementary.pdf]

pharmaceutics

MDPI

Article

Supplementary Materials: Effect of a Fiber D-Limonene-Enriched Food Supplement on Intestinal Microbiota and Metabolic Parameters of Mice on a High-Fat Diet

Maria Chiara Valerii, Silvia Turroni, Carla Ferreri, Michela Zaro, Anna Sansone, Alessandro Dalpiaz, Giada Botti, Luca Ferraro, Renato Spigarelli, Irene Bellocchio, Federica D'Amico and Enzo Spisni

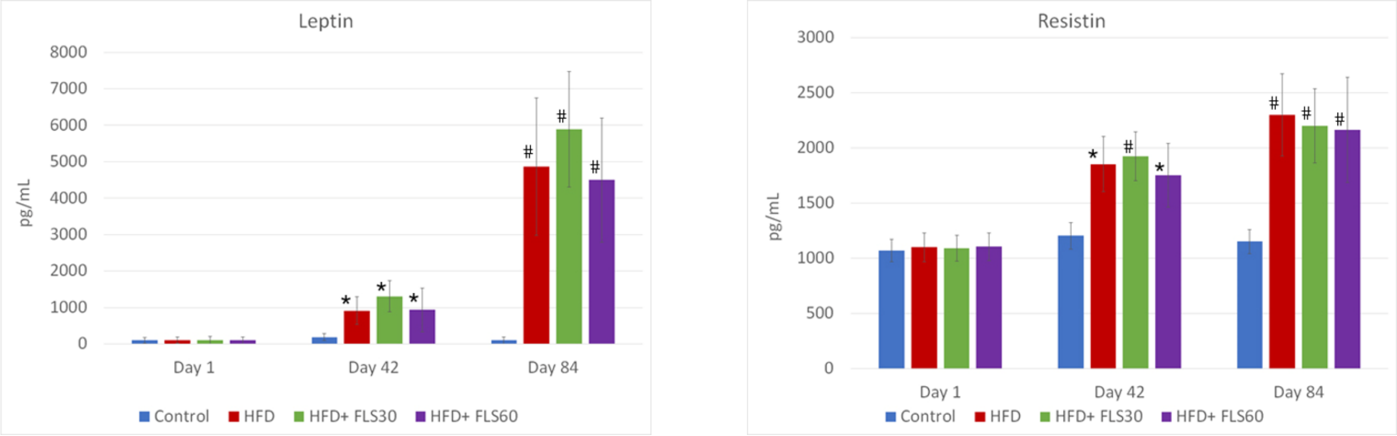

Figure S1. Adipokine levels in Control, HFD and FLS treated mice. High fat diet significantly increased Leptin and Resistin plasma levels over time. FLS at both doses (30 and 60 mg/kg die) did not significantly modify adipokine levels in comparison with those measured in the HFD group. *, $p < 0.05$; #, $p < 0.01$ compared to the Control group. We also measured the Adiponectin plasma levels, but we did not find any significant difference between Control, HFD and FLS groups (not shown).

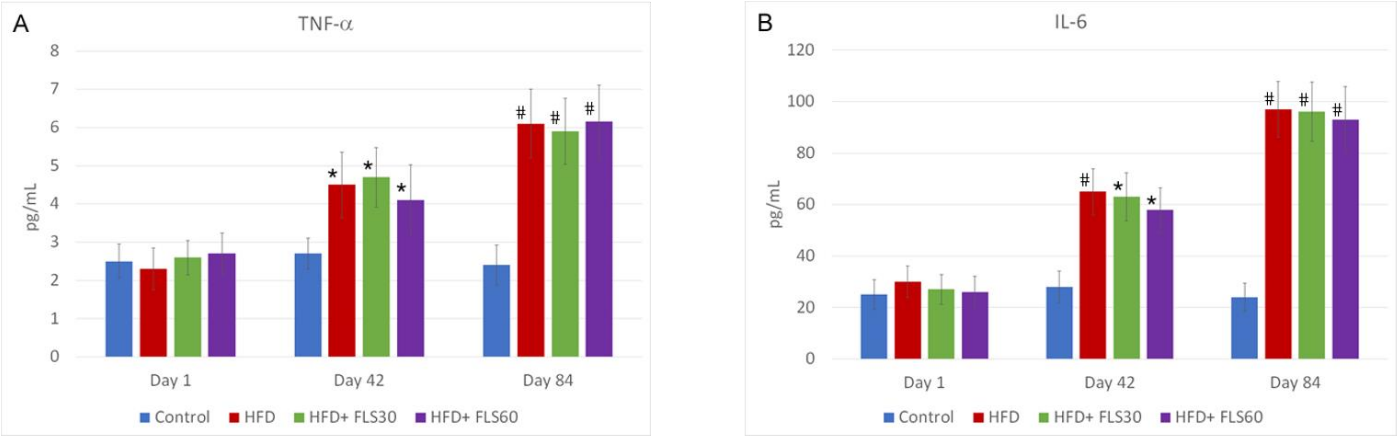

Figure S2. Cytokine levels in Control, HFD and FLS treated mice. High fat diet significantly increased TNF- and IL-6 plasma levels over time. FLS at both doses (30 and 60 mg/kg die) did not significantly modify cytokine levels in comparison with those measured in the HFD group. *, $p < 0.05$; #, $p < 0.01$ compared to the Control group.
